# A novel mass spectrometry-based assay for GSK-3β activity

**DOI:** 10.1186/1471-2091-6-29

**Published:** 2005-12-16

**Authors:** Erin Bowley, Erin Mulvihill, Jeffrey C Howard, Brian J Pak, Bing Siang Gan, David B O'Gorman

**Affiliations:** 1 Cell and Molecular Biology Laboratory, Hand and Upper Limb Centre, Lawson Health Research Institute, St. Joseph's Health Centre, London, Ontario, Canada; 2 Ciphergen Biosystems International Inc., Fremont, California, USA; 3 Department of Surgery, University of Western Ontario, London, Ontario, Canada; 4 Department of Physiology and Pharmacology, University of Western Ontario, London, Ontario, Canada; 5 Department of Medical Biophysics, University of Western Ontario, London, Ontario, Canada

## Abstract

**Background:**

As a component of the progression from genomic to proteomic analysis, there is a need for accurate assessment of protein post-translational modifications such as phosphorylation. Traditional kinase assays rely heavily on the incorporation of γ-P^32 ^radiolabeled isotopes, monoclonal anti-phospho-protein antibodies, or gel shift analysis of substrate proteins. In addition to the expensive and time consuming nature of these methods, the use of radio-ligands imposes restrictions based on the half-life of the radionucleotides and pose potential health risks to researchers. With the shortcomings of traditional assays in mind, the aim of this study was to develop a high throughput, non-radioactive kinase assay for screening Glycogen Synthase Kinase-3beta (GSK-3β) activity.

**Results:**

Synthetic peptide substrates designed with a GSK-3β phosphorylation site were assayed with both recombinant enzyme and GSK-3β immunoprecipitated from NIH 3T3 fibroblasts. A molecular weight shift equal to that of a single phosphate group (80 Da.) was detected by surface enhanced laser desorption/ionization time of flight mass spectrometry (SELDI-TOF-MS) in a GSK-3β target peptide (2B-Sp). Not only was there a dose-dependent response in molecular weight shift to the amount of recombinant GSK-3β used in this assay, this shift was also inhibited by lithium chloride (LiCl), in a dose-dependent manner.

**Conclusion:**

We present here a novel method to sensitively measure peptide phosphorylation by GSK-3β that, due to the incorporation of substrate controls, is applicable to either purified enzyme or cell extracts. Future studies using this method have the potential to elucidate the activity of GSK-3β *in vivo*, and to screen enzyme activity in relation to a variety of GSK-3β related disorders.

## Background

Phosphorylation is believed to be the most common protein post-translational covalent modification and is known to occur in the processing of as many as 1/3 of eukaryotic gene products [1]. That the mammalian genome is predicted to encode as many as 1000 different protein phosphatases and twice as many kinases underlines the importance of protein phosphorylation in cellular function [2,3]. One of the most diverse protein kinases studied to-date is the constitutively active serine/threonine kinase, Glycogen Synthase Kinase-3beta (GSK-3β). Originally identified for its role in the regulation of glycogen metabolism [4], it is now known that GSK-3β plays a key role in cellular processes as diverse as cytoskeletal regulation [5], cell cycle progression [6,7], apoptosis [8], cell fate and specification [9], and transcriptional/translational initiation [10,11]. Therefore, functional kinase activity of GSK-3β is important in a variety of biological and biochemical processes and altered GSK-3β activity can contribute to a number of pathological processes including bipolar mood disorder [12-14], schizophrenia [15], heart disease [16,17], neurodegeneration [18] Alzheimer's disease [11,19] and diabetes mellitus [11,19,20]. Elucidating the direct activity of GSK-3β phosphorylation activity *in vivo *is therefore important in contributing to understanding the molecular basis of a variety of disease states.

Traditionally, kinase assays are performed using radioactive isotopes and scintillation counting for determination of γ-P^32 ^incorporation into a substrate [21]. These methods are relatively insensitive, as they are unsuitable for screening discrete changes in enzyme activity, and are limited by radiation-induced peptide degradation and the short half-life of γ-P^32^. Furthermore, exposure to radioactive isotopes poses a health risk, and thus movement towards a non-radioactive kinase assay is preferable. Existing non-radioactive kinase assays utilize band shifts on non-denaturing polyacrylamide gels and the use of monoclonal antibodies that are indirectly quantified or visualized using Western Blot analysis or immunofluorescence. Such methods are limited by the requirements of specific antibodies for well-characterized phosphorylated residues on a protein of interest, numerous incubation steps, and their time consuming nature when multiple substrates are being screened at once.

This study focuses on the development of a novel, rapid, non-radioactive method of screening GSK-3β activity using surface enhanced laser desorption/ionization time of flight mass spectrometry (SELDI-TOF-MS). This kinase assay utilizes peptide substrates that have been designed with a well-known GSK-3β phosphorylation site based on the translation initiation factor eIF2B [22,23]. GSK-3β has an unusual preference for target proteins that have undergone a previous phospho-priming event, and the enzyme generally recognizes substrates with a Ser-Xaa-Xaa-Xaa-Ser(P) motif [22,24]. The synthetic substrate peptides were prepared with a serine residue at a position equivalent to the GSK-3β phosphorylation site on eIF2B (n), and either an alanine (2B-A), serine (2B-S) or phosphoserine (2B-Sp) at the n+4 position. The phospho-primed serine containing peptide, 2B-Sp is subject to phosphorylation by GSK-3β, while the serine and alanine containing peptides, 2B-S and 2B-A, remain unphosphorylated due to the lack of the phosphoserine residue requisite for GSK-3β phosphorylation. To broaden the applicability of this assay to cell extracts potentially containing priming kinases such as casein kinase-1, the 2B-S peptide has been incorporated as a control substrate that can be converted to 2B-Sp, and subsequently phosphorylated by GSK-3β.

The dual use of SELDI-TOF-MS and GSK-3β target peptides allows for the detection of changes in their molecular weight, or m/z ratio, when subjected to the kinase activity of GSK-3β. Essentially, the target peptides are added in a kinase assay with GSK-3β (either recombinant, or immunoprecipitated) and submitted for mass spectrometric analysis. The peptide samples are spotted on gold (Au) chips, covered with energy absorbing matrix (EAM), inserted into a PBS II ProteinChip^® ^Reader, and desorbed/ionized with a nitrogen-based laser. The components of the sample are then resolved based on time of flight from the chip surface to the detector, which is proportional to their mass to charge ratio (m/z) [25]. Through the selective use of peptide substrates with predetermined mass signatures, SELDI-TOF-MS can identify when a specific peptide substrate has been phosphorylated via a mass/charge (m/z) shift of 80 Da., the molecular weight equivalent of a single phosphate group.

Although matrix assisted mass spectrometric techniques are not particularly quantitative in nature [26], the ability to detect and readily resolve discrete changes in molecular weight makes up for this downfall. Surface enhanced technologies utilizing chromatographic or preactivated surface chips can allow for quantitative measurement. SELDI-TOF-MS was used in this study to determine the phosphorylation status of GSK-3β target peptides through detection of changes in peptide m/z ratio. GSK-3β kinase activity can be derived from this information, an observation of enzyme activity that can only be inferred from less direct assays such as the phosphorylation state of the enzyme itself.

By capitalizing on the ease, sensitivity and reproducibility of SELDI-TOF-MS, a novel non-radioactive, mass spectrometry-based method has been developed to study phosphorylation events. This method can be used to elucidate the signaling activity of a specific kinase *in vivo*, and is limited only by the specificity of the kinase for its substrate peptides [27]. This kinase detection method is highly specific, and accurate protein profiles can be generated from minimal sample volumes in a short amount of time. SELDI-TOF-MS is a relatively new proteomic tool that has been used in the discovery of disease-related biomarkers in carcinomas such as pancreatic ductal adenocarcinoma [28,29], malignant prostate cancer [30], breast cancer [31], and ovarian neoplasms [32]. Under the premise of this type of GSK-3β activity detection, it is also possible to deduce the kinase activity of GSK-3β in more complex cellular signaling systems.

## Results

### SELDI-TOF-MS analysis of GSK-3β target substrates

**Figure 1:**
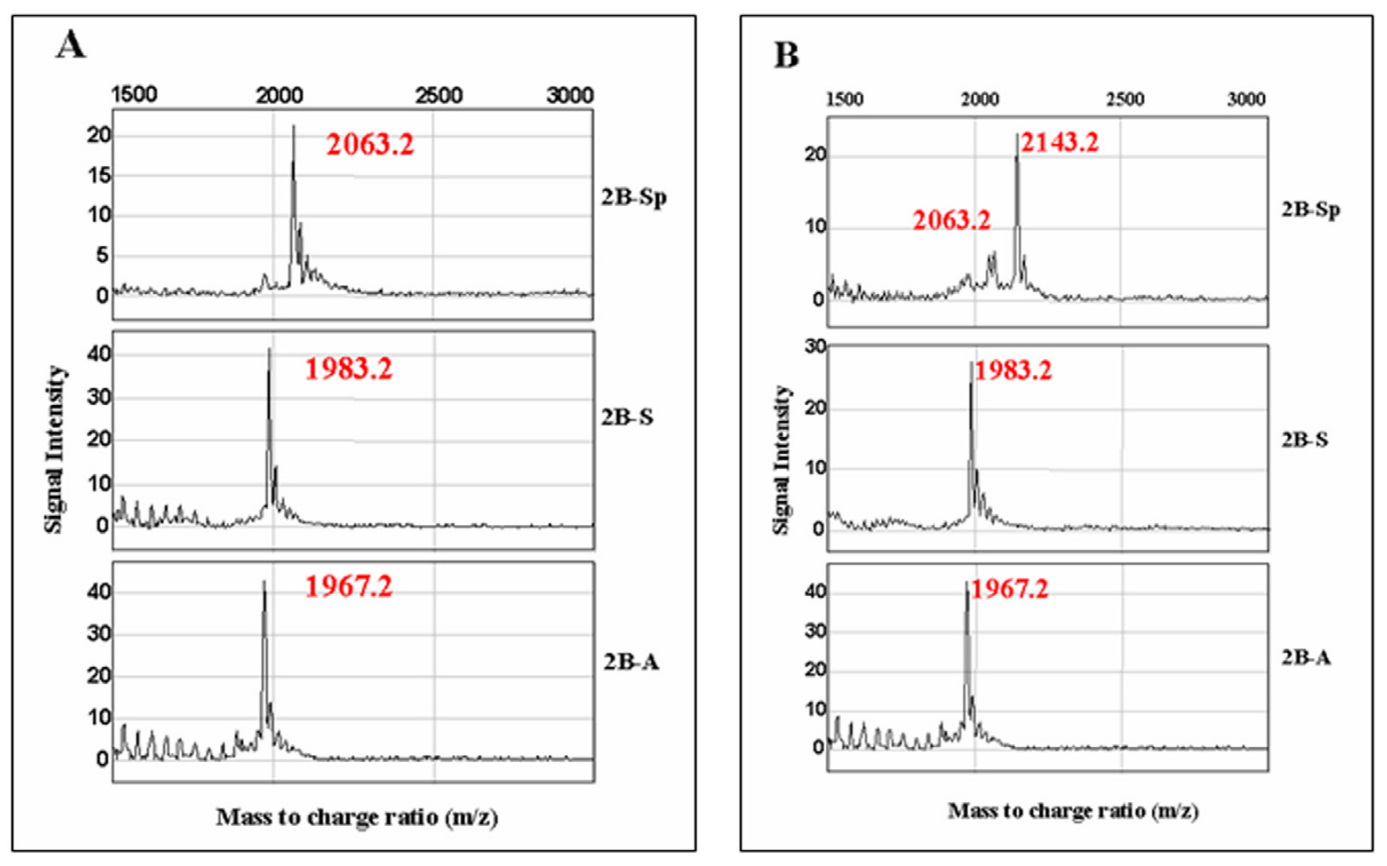
**SELDI-TOF-MS analysis of GSK-3β target peptides. (A) **GSK-3β target peptide substrates and representative SELDI-TOF-MS analysis spectra. SELDI-TOF-MS representative spectra of peptides 2B-Sp, 2B-S, and 2B-A reveal a single m/z peak corresponding to their natural molecular weights (2063.2 Da., 1983.2 Da., and 1967.2 Da., respectively). **(B) **SELDI-TOF-MS spectra of target peptides subjected to a kinase assay with recombinant GSK-3β. Each target peptide substrate was incubated with 6.25 ng of recombinant GSK-3β enzyme, a magnesium/ATP kinase reaction mixture, and incubated for 20 minutes at 37°C. GSK-3β kinase activity was detected as an 80 Da. shift in peptide m/z ratio.

Parent m/z ratio peaks of GSK-3β target synthetic peptides, 2B-Sp, 2B-S, and 2B-A were determined by preparing samples containing untreated peptide substrates for SELDI-TOF-MS analysis. Native molecular weights were obtained and calibrated in accordance with manufacturer m/z ratio read-outs of each of 2B-Sp, 2B-S, and 2B-A peptides. The m/z ratio of 2B-Sp, 2B-S, and 2B-A peptides were 2063.2 Da., 1983.2 Da., and 1967.2 Da., respectively (Figure 1A).

### Recombinant GSK-3β induces a shift in the m/z peak of 2B-Sp

Mass to charge ratios of peptide substrates 2B-Sp, 2B-S, and 2B-A incubated with recombinant GSK-3β were analyzed using SELDI-TOF-MS. A shift of peptide m/z ratio of 2063.2 Da. to 2143.2 Da. was detected in 2B-Sp kinase assay samples only, corresponding to the addition of a single phosphate group of 80 Da. (Figure 1B upper). The m/z ratio for 2B-S and 2B-A peptides remained at their parent molecular weight of 1983.2 Da. and 1967.2 Da., respectively (Figure 1B).

### The m/z ratio shift of 2B-Sp is GSK-3β dose-dependent

**Figure 2:**
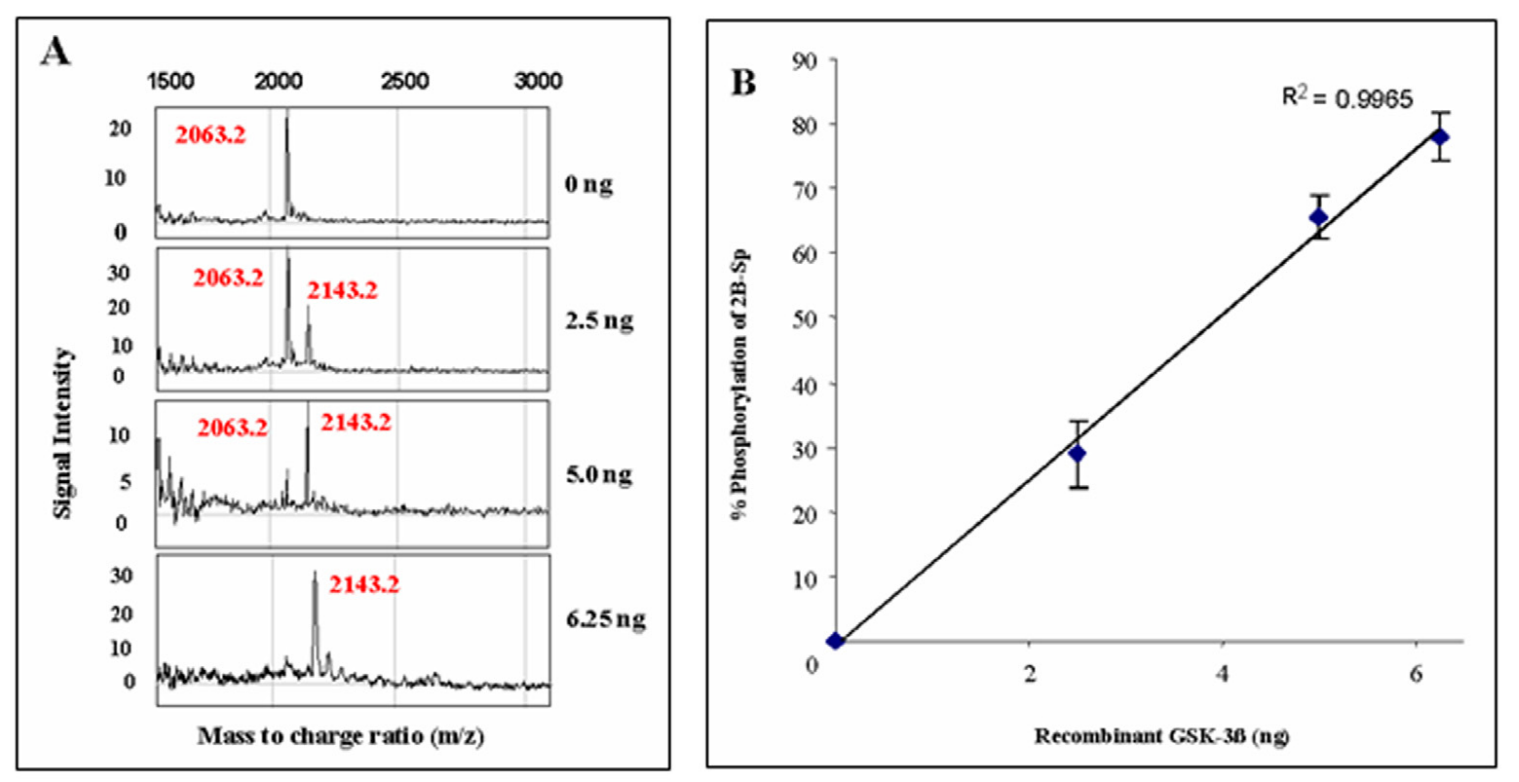
**A dose-dependent shift in 2B-Sp m/z ratio in response to increasing amounts of recombinant GSK-3β**. 125 ng of 2B-Sp was added to kinase assays with increasing amounts of recombinant GSK-3β (0 – 6.25 ng). **(A) **Raw data is presented here as a SELDI-TOF-MS m/z read-out. **(B) **Enzyme activity was measured as percent 2B-Sp phosphorylation, and was determined through the ratio calculation of the 2143.2 Da. peak intensity to the total signal intensity values of all peptide present in the sample (2063.2 Da. and 2143.2 Da. peaks combined). Pooled data are plotted as mean values +/- SEM (n = 3) at each data point.

A dose-response assay determined that the 80 Da. increase in m/z ratio of the 2B-Sp peptide corresponded to amount of recombinant GSK-3β used in each assay (Figure 2A). Kinase assays were performed with 125 ng of the 2B-Sp peptide and increasing amounts of recombinant GSK-3β (0 – 6.25 ng). Percent 2B-Sp phosphorylation increased from 0% at 0 ng of GSK-3β to almost 80% when 6.25 ng of recombinant GSK-3β was used in the assay (Figure 2B).

### 2B-Sp peptide m/z shift is sensitive to GSK-3β inhibition by lithium chloride

**Figure 3:**
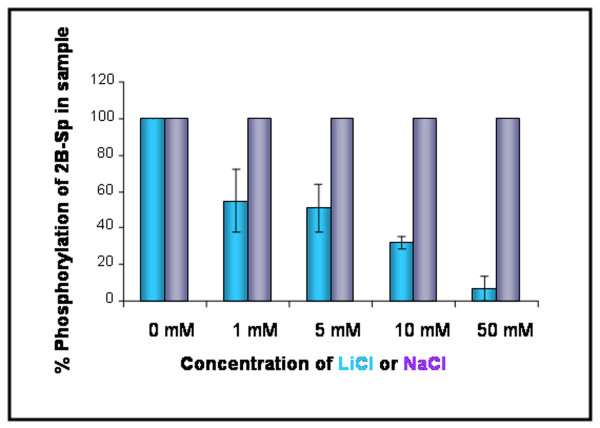
**2B-Sp m/z ratio shifts in response to varying amounts of lithium chloride, and sodium chloride**. GSK-3β specificity for the 2B-Sp target peptide was examined in a kinase assay with lithium chloride (LiCl, green bars, n = 3) a well-known inhibitor of GSK-3β, and sodium chloride (NaCl, purple bars, n = 3), a salt with no known GSK-3β inhibitory characteristics. Increasing amounts of LiCl (0–50 mM), or NaCl (0–50 mM) were added to kinase assays containing 125 ng of 2B-Sp peptide, and 6.25 ng of recombinant GSK-3β. M/z ratios were subsequently analyzed using SELDI-TOF-MS. Pooled data for LiCl assays are represented as mean +/- SEM values. NaCl assays are represented as mean values.

Kinase assays were performed with LiCl, an established GSK-3β inhibitor, and NaCl, a salt with no known inhibitory action on GSK-3β activity. Kinase assays were prepared with 2B-Sp peptide, recombinant GSK-3β, and increasing concentrations (0 – 50 mM) of LiCl or NaCl (Figure 3). As the concentration of LiCl increased, there was a corresponding decrease in the 2143.2 Da. 2B-Sp peak, and a marked increase in the signal intensity of the parent 2B-Sp 2063.2 Da. peptide peak (Figure 3). Percent phosphorylation ratios decreased from 100% at 0 mM LiCl to 6.64% at 50 mM LiCl (Figure 3). In contrast, NaCl had no effect on the kinase activity of GSK-3β, as an 80 Da. shift in 2B-Sp m/z ratio was evident regardless of the concentration of NaCl used in the assay (Figure 3).

### Radiolabeled kinase assays confirm recombinant GSK-3β phosphorylation of 2B-Sp target peptide

**Figure 4:**
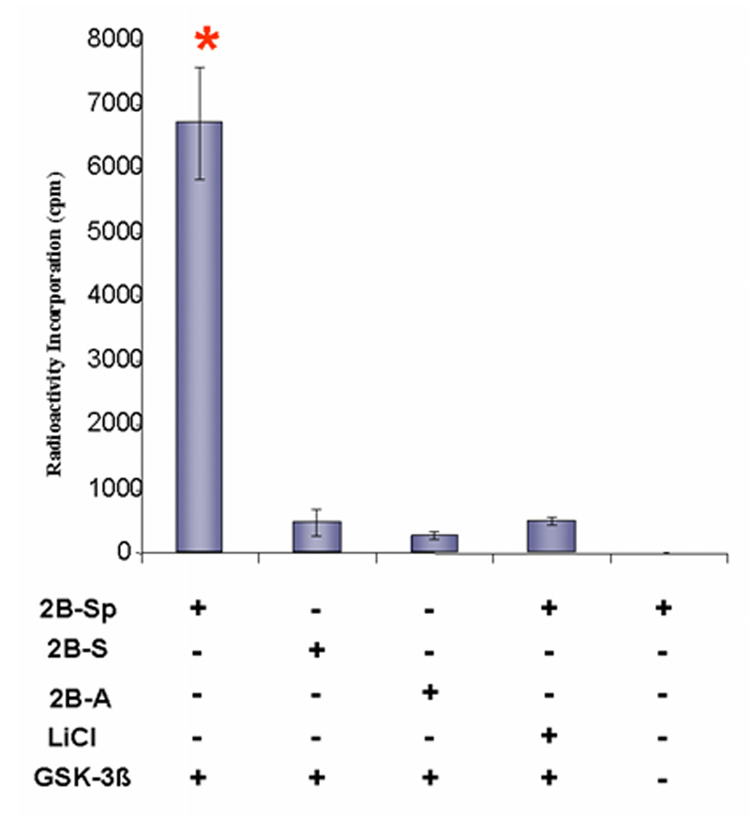
**Traditional kinase assay: γ-^32^P incorporation into target and control peptides**. Kinase assays were prepared with target peptides 2B-Sp, 2B-S, 2B-A, and γ-^32^P labeled ATP. Incorporation of γ-^32^P into each peptide was quantified in counts per minute (cpm) by liquid scintillation counting. Assays were performed with 6.25 ng of recombinant GSK-3β, and in the absence or presence of 50 mM LiCl. An assay devoid of GSK-3β enzyme in the presence of 2B-Sp controlled for background radioactivity readings. Incorporation of radioactivity in cpm units were plotted as means +/- SEM (n = 9). * denotes statistical significance by ANOVA and Tukey's post-hoc analysis (p < 0.05).

A traditional kinase assay was performed to detect recombinant GSK-3β-mediated incorporation of radiolabeled [γ-P^32^] ATP into the peptide substrates described previously. Once background radioactivity readings derived from reactions without recombinant GSK-3β were normalized, minimal radioactivity readings were detected in assays containing the 2B-S and 2B-A target peptides (620.78 +/- 206.92 cpm and 192.95 +/- 64.31 cpm, respectively) relative to γ-P32 incorporation into 2B-Sp (6701.66 +/- 873.56 cpm) (Figure 4). Very low levels of substrate phosphorylation were also observed in the presence of LiCl (510.61 +/- 66.06 cpm). A one-way analysis of variance (ANOVA) was conducted for radioactivity incorporation into GSK-3β target peptides. Following the significant ANOVA [F(3,8) = 16.16, p < 0.01, power = 0.858], Tukey post-hoc tests (p < 0.05) showed that the 2B-Sp target peptide group had significantly more radioactivity incorporation than the other groups tested. * Indicates a significant mean difference in radioactivity incorporation.

### Determination of GSK-3β activity in NIH 3T3 cell lysates

**Figure 5:**
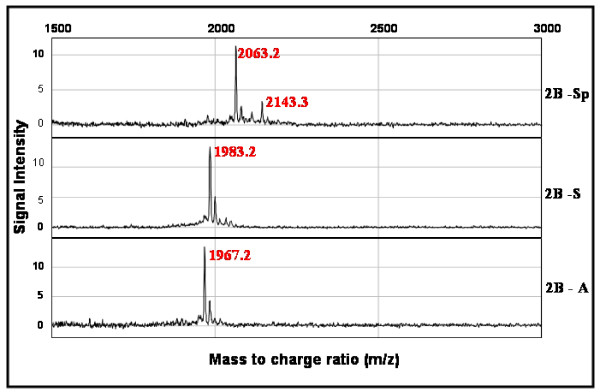
**GSK-3β activity in immunoprecipitated NIH 3T3 cell lysates determined by SELDI**. GSK-3β activity was determined as described in the text from immunoprecipitated NIH 3T3 cell lysates. SELDI-TOF-MS representative spectra displaying the mass/charge ratio (m/z) and signal intensities for the 2B-Sp, 2B-S and 2B-A peptides are shown. The mass change from 2063.2 Da. to 2143.3 Da. in the 2B-Sp chip is consistent with the addition of one phosphate group (80 Da.). This experiment was replicated three times and the area under the 3143.3 Da. peak was quantitated relative to the area under the 2063.2 Da. peak indicating 23.0% +/- 3.5% phosphorylation of the 2B-Sp peptide.

GSK-3β activity was determined from immunoprecipitated NIH 3T3 cell lysates as described above for recombinant GSK-3β. As shown in figure 5, immunoprecipitated NIH 3T3 cell lysates displayed two peaks at 2063.2 Da. and 2143.2 Da. (Figure 5). The 2B-S and 2B-A peptides remained at original molecular weights of 1983.2 Da. and 1967.2 Da. in all samples tested.

## Discussion

Although genomic research has made remarkable discoveries in the last few decades, there are still many unsolved mysteries regarding the actual protein products of the genetic code. As it is estimated that approximately 50% of all proteins undergo one or more post-translational modifications that alter both their structure and function, it is clear that meaningful predictions of protein status cannot be made by genomic research alone [33]. Protein phosphorylation is a post-translational modification that is essential for many cellular pathways, and for this reason has become the focus of recent proteomic-based research. Phosphoproteomics has stepped into a light of its own with many advances being made in mass spectrometric techniques which allow for rapid, high-throughput protein detection and resolution [34]. With such advances in proteomic analysis, it is now possible to explore intracellular phosphorylation events and the role they play in complex cellular signaling systems.

GSK-3β generally has two sites of substrate recognition which can be divided into two different classes: substrates which require a phospho-priming event, and those which do not [35]. Previous studies have determined that synthetic peptides designed with a phospho-primed serine four residues downstream of a serine residue are the most effective substrates for assaying GSK-3β activity *in vitro *[22,35].

The eIF2B sequence-like peptide 2B-Sp and recombinant GSK-3β were used to optimize the conditions needed for the assay, including sample preparation, and SELDI-TOF-MS analysis. It was determined that 6.25 ng of recombinant GSK-3β resulted in a 80 Da. shift in molecular weight for 100% of the target serine residue in the 2B-Sp peptide and that a stepwise reduction in recombinant GSK-3β to 2.5 ng resulted in a dose-response reaction with respect to phosphorylation status of the peptide (Figure 2). Furthermore, assays with 6.25 ng of recombinant GSK-3β and control peptides 2B-S and 2B-A did not produce an 80 Da. shift in m/z ratio (Figure 1B). These results indicate that recombinant GSK-3β kinase activity was unable to phosphorylate the 2B-A control peptide and that, in the absence of priming kinases, the SB-S peptide was also an effective control for specific GSK-3β phosphorylation (Figure 1B).

To further support our findings that the 80 Da. shift in m/z ratio of the 2B-Sp peptide was due to the addition of a phosphate moiety and the result of GSK-3β activity, inhibitory assays were performed. An established inhibitor of GSK-3β, lithium chloride (LiCl), and a non inhibitor control salt, sodium chloride (NaCl) were added to subsequent assays. Increasing concentrations of LiCl inhibited the 80 Da. shift in molecular weight in a dose-dependent manner (Figure 3). In contrast, NaCl was shown to be ineffective in altering the m/z ratio of the target serine on the 2B-Sp peptide (Figure 3), confirming LiCl specific inhibition of GSK-3β kinase activity.

Although an 80 Da. shift in molecular weight of the 2B-Sp peptide is consistent with a phosphorylation event catalyzed by GSK-3β, traditional γ-P^32 ^assays were performed to confirm the incorporation of a phosphate group into the target peptide. Scintillation counting confirmed the incorporation of γ-P^32 ^into the 2B-Sp peptide but not the 2B-S and 2B-A controls (Figure 4). Similarly, the addition of LiCl inhibited GSK-3β kinase activity on the 2B-Sp peptide (Figure 4).

Having validated our method of screening GSK-3β activity by showing specificity for the target peptide used and that this method is capable of detecting changes in enzyme activity, we further validated this system for use in a biological model. Serum-starved NIH 3T3 fibroblasts were lysed and GSK-3β was immunoprecipitated. Enzyme activity was assessed in the same manner as described previously for recombinant purified GSK-3β. As shown in figure 5, immunoprecipitated cell lysates from fibroblasts display a large 2063.2 Da. peak and a smaller but clearly detectable peak at 2143.2 Da. corresponding to non-phosphorylated and phosphorylated 2B-Sp peptide respectively. Relative quantitation of areas under the peaks from three replicate experiments indicated that 23.0% +/- 3.5% of the 2B-Sp peptide had been phosphorylated. This demonstrates that this assay system is applicable to biological systems where the sensitive detection of changes in GSK-3β activity is required. In contrast, the 2B-S peptide control remained at its original molecular weight of 1983.2 Da. indicating that there was no detectable contamination from priming kinases in the immunoprecipitated lysates. The 2B-A peptide remained at original molecular weight of 1967.2 Da. in all samples indicating that there were no other, less specific kinases contaminating the immunoprecipitates and that both 2B-A and 2B-S peptides are effective controls for this assay.

## Conclusion

The kinase activity detection method developed in this study has applications outside of its use for screening GSK-3β activity. The versatility of this method lies in the simplicity of its design: employing the use of mass spectrometry to detect small changes of molecular weight in peptide substrates specific for a protein kinase of interest. If target peptide substrates can be synthesized with a sequence specific for the protein kinase (or phosphatase) of interest, this method is applicable to virtually any signaling pathway in which kinase activity and phosphorylation play a role.

This report describes in general terms the components required and sensitivity of this SELDI-TOF assay for GSK3β activity. It is emphasized that cell-type specific optimization is required to utilize this assay system. Immunoprecipitation of cell lysates is a necessary and essential component of this assay as it facilitates the measurement of GSK3β activity specifically and, ideally, in the absence of interfering factors such as priming kinases. Optimal conditions for immunoprecipitation are necessary to achieve target specificity as well as reproducible and sensitive detection of changes in GSK3β activity.

The demonstration of altered GSK-3β kinase activity in a variety of pathologies related to fibroproliferative disease lead to its selection for this study. Not only is GSK-3β activity implicated in a variety of developmental, cell signaling, and regulatory mechanisms [36], dysfunctional GSK-3β activity is now being explored in Alzheimer's disease [37], non-insulin dependent diabetes mellitus (NIDDM) [38], and in our laboratory, Dupuytren's contracture, a fibroproliferative disease whose molecular pathology is not presently understood. With the development of this SELDI-TOF-MS based assay for screening GSK-3β kinase activity, future studies can be directed at elucidating the role of this protein kinase in a variety of disease states in hopes of characterizing their molecular pathways.

## Methods

### Synthetic peptide substrates

Peptides used in this study (AnaSpec Incorporated, San Jose, CA) were;

• Biotin-Arg-Arg-Ala-Ala-Glu-Glu-Leu-Asp-**Ser-Arg-Ala-Gly-Ser^PO4^**-Pro-Gln-Leu-OH (2B-Sp, molecular weight 2063.2 Da.)

• Biotin-Arg-Arg-Ala-Ala-Glu-Glu-LeulAsp-**Ser-Arg-Ala-Gly-Ser**-Pro-Gln-Leu-OH

(2B-S, molecular weight 1983.2 Da.)

• Biotin-Arg-Arg-Ala-Ala-Glu-Glu-Leu-Asp-**Ser-Arg-Ala-Gly-Ala**-Pro-Gln-Leu-OH

(2B-A, molecular weight 1967.2 Da.)

Peptide substrates were diluted with HPLC grade water to working dilutions and stored at -80°C for later use in subsequent kinase assays.

### Cell culture

NIH 3T3 cells were cultured in Dulbecco's Modified Eagle's Medium (DMEM) with 10% Bovine calf serum and fresh 2 mM glutamine (Invitrogen Canada Inc. Burlington, Ontario). Cells were cultured in 6 well trays and, when 80% confluent, were serum starved in DMEM for 12 hours.

### Protein extraction and immunoprecipitation

Whole-cell lysates were isolated using RIPA buffer with protease inhibitors (Sigma-Aldrich, Milwaukee, WI) and total protein concentrations were determined by BCA (Pierce Biotechnology, Rockford, IL). GSK-3β was immunoprecipitated using anti- GSK-3β (BD Biosciences, Mississauga, ON) using standard protocols. In brief, 50–200 μg of cell lysate was cleared with 20 μl of Protein A/G Plus agarose beads (Santa Cruz Biotechnology, Santa Cruz, CA), on a rotator at 4°C for 1 hr. The suspension was centrifuged briefly, the supernatant was separated and 2 μg of GSK-3β antibody was added and rotated at 4°C for 1 hr. 20 μL of Protein A/G Plus Agarose beads were added and the suspension was rotated at 4°C overnight. The suspension was centrifuged briefly and the supernatant discarded. The bead/antibody/GSK-3β complex was washed 3 times with 40 μl of magnesium/ATP cocktail at 4°C immediately prior to the kinase reaction.

### Kinase assays

The activity of recombinant GSK-3β (Upstate, Lake Placid NY) or immunoprecipitated GSK-3β derived from NIH 3T3 fibroblasts was measured in a kinase assay with 125 ng of peptide (2B-Sp, 2B-S or 2B-A) and 6.25 ng of either recombinant or immunoprecipitated GSK-3β. The kinase reaction mixture consisted of 5 μl of 5 × Reaction Buffer (40 mM MOPS pH 7.0, 1 mM EDTA), 10 μl of magnesium/ATP cocktail (Final concentrations of 15 mM MgCl_2_, 100 μM Adenosine tri-phosphate (ATP), 4 mM 3-(N-Morpholino) propanesulfonic acid (MOPS) pH 7.2, 1 mM Ethylene glycol-bis (2-aminoethylether)-N,N,N',N'-tetraacetic acid (EGTA), 200 μM sodium orthovanadate (NaVO_3_) and 200 μm dithiothreitol (DTT)). The kinase assay was incubated for 20 minutes at 37°C. GSK-3β activity was subsequently analyzed by SELDI-TOF-MS.

Traditional kinase assays were performed using 125 ng of peptide, 2 μl ATP cocktail (1.25 nmoles cold ATP, 1.875 × 10^6 ^cpm [γ-P^32^] ATP (MP Biomedicals, Irvine CA), 5 μl of 5 × reaction buffer, and 10 μl of magnesium/ATP cocktail, as described above. 6.25 ng of recombinant GSK-3β was used in the radio-labeled reaction mixture to a final volume of 25 μl in HPLC grade water (Sigma-Aldrich, Milwaukee, WI). The [γ-P^32^] labeled peptide was blotted on p81 phosphocellulose paper (Whatman) and washed 5× with 100 mM phosphoric acid (Sigma-Aldrich, Milwaukee, WI). Incorporation of γ-P^32 ^was quantified in counts per minute (cpm) by liquid scintillation (Fischer Scientific, Fair Lawn, NJ).

### SELDI-TOF mass spectrometry

Kinase assay samples were acidified for ProteinChip^® ^processing with 10% trifluoroacetic acid (TFA). Equilibrated Millipore Ziptip^® ^C18 columns (Millipore Corporation Bedford, MA) were used to bind, wash, and elute the kinase assay sample as described by the manufacturer. Elution samples were spotted on gold (Au) ProteinChip^® ^Arrays (Ciphergen Biosystems Inc. Fremont Ca.), dried, and covered with a saturated solution of α-cyano-4-hydroxy cinnamic acid (CHCA, Sigma, St. Louis, MO) energy absorbing molecule. Samples were read using the PBS-II ProteinChip^® ^Reader and the resulting data were analyzed using the ProteinChip^® ^Software v3.2 for changes in mass to charge (m/z) ratios.

## Authors' contributions

EB and EM carried out kinase assays, cell culture and SELDI analysis, as well as interpretation of the data and first drafting of the manuscript. JCH, BSG and DBO participated in study conception and design. BJP carried out supplemental SELDI analysis and provided technical advice and trouble-shooting expertise. DBO designed the cell culture experiments. DBO and BSG coordinated the entire project, performed final interpretation of the data and completed the manuscript. All authors read and approved the final manuscript.
